# Preparation of a Novel Oat *β*-Glucan–Chromium(III) Complex and Its Hypoglycemic Effect and Mechanism

**DOI:** 10.3390/molecules29091998

**Published:** 2024-04-26

**Authors:** Pengshou Li, Yunlu Wang, Xiaoting Wang, Rui Li, Kaihui Wang, Yu Jiang, Mingyuan Zhang, Chuhan Huang, Qixiang Ma, Jian Sun, Jianye Quan

**Affiliations:** 1 College of Food and Drug, Luoyang Normal University, Luoyang 471934, China; lps0925@lynu.edu.cn (P.L.); 18336700828@163.com (Y.W.); 13839304086@163.com (X.W.); l2955531963@163.com (R.L.); 13939203273@163.com (K.W.); 15294795805@163.com (Y.J.); 13213529280@163.com (M.Z.); 17345006933@163.com (C.H.); 2 Cancer Institute, Fudan University Cancer Hospital and Cancer Metabolism Laboratory, Institutes of Biomedical Sciences, Fudan University, Shanghai 200032, China; maqixiang@fudan.edu.cn; 3 Experimental Research Center, China Academy of Chinese Medical Sciences, Beijing 100700, China

**Keywords:** oat *β*-glucan, chromium(III) complex, structure, hypoglycemic activity, hypoglycemic mechanism

## Abstract

This study synthesized a novel oat *β*-glucan (OBG)-Cr(III) complex (OBG-Cr(III)) and explored its structure, inhibitory effects on α-amylase and α-glucosidase, and hypoglycemic activities and mechanism in vitro using an insulin-resistant HepG2 (IR-HepG2) cell model. The Cr(III) content in the complex was found to be 10.87%. The molecular weight of OBG-Cr(III) was determined to be 7.736 × 10^4^ Da with chromium ions binding to the hydroxyl groups of OBG. This binding resulted in the increased asymmetry and altered spatial conformation of the complex along with significant changes in morphology and crystallinity. Our findings demonstrated that OBG-Cr(III) exhibited inhibitory effects on α-amylase and α-glucosidase. Furthermore, OBG-Cr(III) enhanced the insulin sensitivity of IR-HepG2 cells, promoting glucose uptake and metabolism more efficiently than OBG alone. The underlying mechanism of its hypoglycemic effect involved the modulation of the c-Cbl/PI3K/AKT/GLUT4 signaling pathway, as revealed by Western blot analysis. This research not only broadened the applications of OBG but also positioned OBG-Cr(III) as a promising Cr(III) supplement with enhanced hypoglycemic benefits.

## 1. Introduction

Diabetes mellitus (DM) is a chronic metabolic disease resulting from a combination of genetic and environmental factors [1]. Diabetes mellitus can be categorized into different types, including type I diabetes mellitus, type II diabetes mellitus, gestational diabetes mellitus, and special type diabetes mellitus [2]. Diabetes can lead to complications such as diabetic nephropathy, diabetic eye disease, diabetic foot ulcers, diabetic heart disease, and diabetic peripheral neuropathy [3]. The use of existing diabetes medications may have adverse effects, such as weight gain and acute cholestatic hepatitis [4]. Consequently, the development of novel and safe hypoglycemic products is crucial.

Chromium exists in trivalent and hexavalent forms in nature. Trivalent chromium is the most prevalent form in living organisms, and it can stimulate the metabolism of carbohydrates, lipids, proteins, and minerals [5]. Studies have demonstrated that chromium(III) can reduce insulin resistance, improve glucose tolerance, increase insulin receptor levels, and enhance insulin activity [6,7,8,9]. Additionally, the chemical form of chromium affects its absorption, with organic chromium being more readily absorbed by the human body than inorganic chromium [10].

Currently, natural active polysaccharides are increasingly recognized for their role in the treatment of diabetes [11]. Oat *β*-glucan (OBG) is a kind of soluble dietary fiber [12]. It mainly exists in the cell wall of the oat endosperm and aleurone layer, and it is a non-starch polysaccharide linked by glucose via *β*-(1→3) and *β*-(1→4) glycosidic bonds [13]. OBG had various physiological effects, including hypoglycemic activity [14,15,16]. The consumption of oats has been shown to reduce the risk of heart cerebrovascular disease, diabetes, and high blood pressure. The hypoglycemic effect of oats has also been approved by the U.S. Food and Drug Administration (FDA) [17]. Oat intake significantly affects blood lipids and postprandial blood glucose levels, and oat *β*-glucan plays a substantial role in this relationship [18]. Other studies have indicated that the hypoglycemic mechanisms of oat *β*-glucan includes an increase in gut microbiota abundance and diversity and an increase in the firmicutes/bacteroidetes ratio [19]. It has also been shown to improve intestinal mucosal mechanical and biological barriers in type 2 diabetic rats [20]. Oat *β*-glucan can also prevent and reduce the occurrence and development of diabetes and its complications by inhibiting carbohydrate absorption, promoting liver glycogen synthesis, and enhancing the body’s antioxidation [21].

Polysaccharide–chromium(III) complexes have exhibited promising hypoglycemic activity. These complexes combine the benefits of polysaccharides and chromium(III), offering an improved control of diabetes [22]. Research demonstrated that a Momordica charantia L. polysaccharide–chromium(III) complex possessed excellent anti-hyperglycemic activity and safety [23]. Ye et al. reported that a sulfated rhamnose polysaccharide–chromium(III) complex could improve glucose metabolism by activating the IR/IRS-2/PI3K/PKB/GSK-3*β* signaling pathway and enhancing glucose transport through insulin signaling cascade-induced GLUT4 translocation [24]. Li et al. reported that a Ganoderma lucidum polysaccharide–chromium(III) complex exhibited remarkable hypoglycemic and hypolipidemic activities [25]. Zhang et al. discovered that a novel pumpkin peel polysaccharide–chromium(III) complex exerted hypoglycemic effects via the AMPK/GSK-3β signaling pathway [26]. Xiu et al. reported that a sweet corncob polysaccharide–chromium(III) complex had greater hypoglycemic potential compared to sweet corncob polysaccharide [27]. OBG has a range of health benefits, including lowering cholesterol, having an antioxidant activity, lowering blood sugar, and regulating gut flora [12]. Many clinical studies and oral experiments showed that OBG has good hypoglycemic activity and safety in the human body [28,29,30], and has been approved by FDA, while other polysaccharides’ hypoglycemic activity research mostly stayed at the level of cell or animal experiments. In addition, since natural polysaccharides are derived from plants, animals, and microorganisms in nature, the process of separation and purification is usually time-consuming and cumbersome, and cannot achieve the desired purity and yield; therefore, most of them cannot be used as raw materials in the production of polysaccharide–chromium complexes [31]. Compared with other polysaccharides, OBG has obvious and widely recognized hypoglycemic activity, and its preparation process has been industrialized and easy to obtain in large quantities. Therefore, based on the advantages of OBG, it would be interesting to investigate whether OBG can form complexes with Cr(III) and whether the resulting complexes exhibit an enhanced hypoglycemic effect. 

Therefore, in this study, we used OBG as a ligand to form a new chromium complex, OBG-Cr(III). Various analytical methods confirmed the structure of the complex. The hypoglycemic activity and mechanism of the complex were studied in vitro, which may provide a reference for the utilization of oat *β*-glucan and the research of polysaccharide–chromium complexes. This study will provide a reference for the application and development of OBG and the research of polysaccharide–chromium complexes. The results will also guide the development of promising Cr(III) supplements for the prevention and treatment of diabetes.

## 2. Results

### 2.1. Synthesis of OBG-Cr(III)

**Figure 1:**
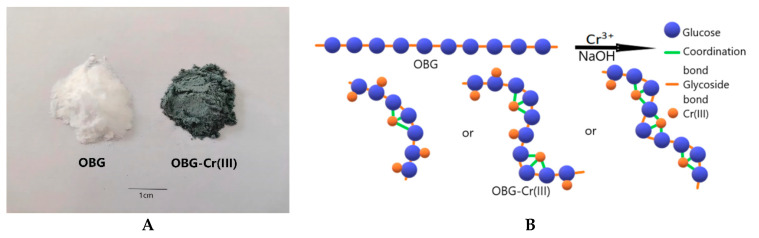
OBG and OBG-Cr(III) ((**A**) sample status; and (**B**) schematic with proposed structure).

By adopting the method in Section 4.2, we prepared and purified OBG-Cr(III). The complex was a gray-green powder (as shown in Figure 1A), which had good water solubility and was insoluble in most organic solvents. The schematic with propose structure of OBG-Cr(III) is shown in Figure 1B. The yield of the complex was 43.5%.

### 2.2. Structural Characterization of OBG-Cr(III)

#### 2.2.1. Molecular Weight Analysis of OBG and OBG-Cr(III)

**Figure 2:**
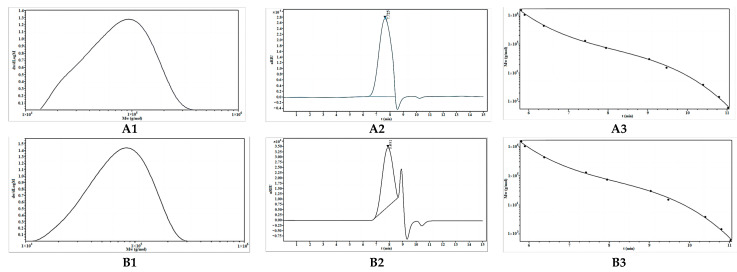
Determination of molecular weight of OBG and OBG-Cr(III) ((**A1**) the molecular weight distribution of the OBG; (**A2**) the GPC chromatogram of the OBG; (**A3**) GPC column correction profile; (**B1**) the molecular weight distribution of the OBG-Cr(III); (**B2**) the GPC chromatogram of the OBG-Cr(III); and (**B3**) GPC column correction profile).

As can be seen from Figure 2, the molecular weight of OBG was 8.894 × 10^4^ Da, and the molecular weight of OBG-Cr(III) was 7.736 × 10^4^ Da (the left main peak was taken as the object of study, and it was separated using Sephadex gel column chromatography). The reaction condition of chromium and oat *β*-glucan was under alkaline and at 70 °C, which would result in the hydrolysis of oat *β*-glucan to some extent, resulting in the decrease in the molecular weight of the chromium complex. 

#### 2.2.2. Chromium Content Analysis of OBG-Cr(III)

The content of trivalent chromium in OBG-Cr(III) was determined by means of ICP-MS. The results showed that the content of Cr(III) in complex was 10.87%. The relevant data are shown in Table 1.

#### 2.2.3. Fourier-Transform Infrared (FT-IR) Spectrum Analysis

**Figure 3:**
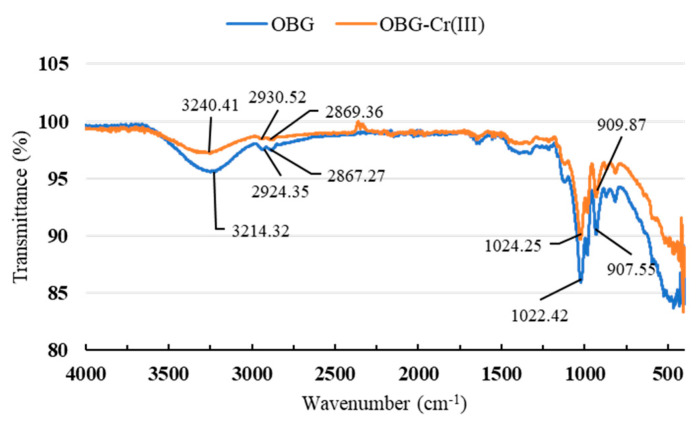
FT-IR spectra of OBG and OBG-Cr(III) complex.

In order to further characterize the structure of OBG-Cr(III), the FT-IR spectra of OBG and OBG-Cr(III) were analyzed. As can be seen from Figure 3, both OBG and OBG-Cr(III) had absorption peaks of saccharides, with OBG at 3214.32 cm^−1^ and OBG-Cr(III) at 3240.41 cm^−1^ being the stretching vibration peaks of the hydroxyl groups of saccharides [23,25,32]. The change in the wavenumber of the peak indicated that Cr(III) coordinated with the hydroxyl group of OBG to form the chromium complex. The weak absorption peaks of OBG at 2924.35 cm^−1^, 2867.27 cm^−1^ and OBG-Cr(III) at 2930.52 cm^−1^, 2869.36 cm^−1^ were C-H stretching vibration peaks in methylene [23,25,33]. The wavenumber of the peak did not change greatly, indicating that the formation of the complex had little effect on the methylene group. The absorption peaks of OBG at 1022.42 cm^−1^ and OBG-Cr(III) at 1024.25 cm^−1^ indicated that both OBG and OBG-Cr(III) contained pyranoside [23]. The absorption peaks of OBG at 907.55 cm^−1^ and OBG-Cr(III) at 909.87 cm^−1^ were the characteristic absorption peaks of *β*-D-pyranose C-H formation vibration [34]. According to FT-IR spectra, most characteristic peaks of OBG-Cr(III) were similar to those of OBG, indicating that the basic skeleton of OBG remained unchanged after the formation of the complex.

#### 2.2.4. Ultraviolet and Visible (UV-VIS) Spectrum Analysis

**Figure 4:**
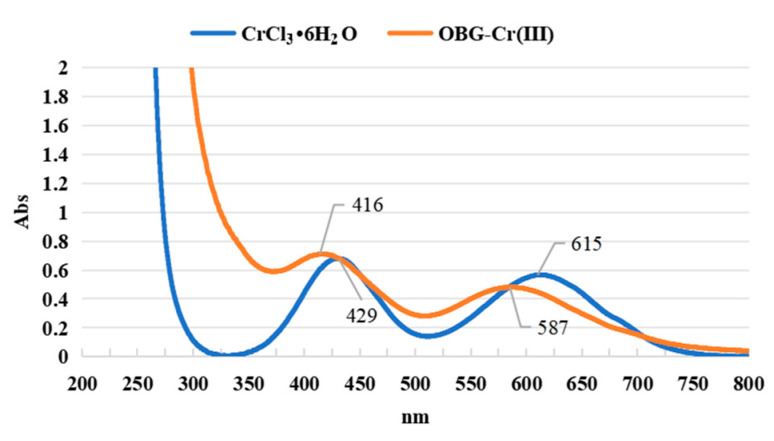
UV-VIS spectroscopy.

As shown in Figure 4, for CrCl_3_·6H_2_O, the two broad absorption peaks at 429 nm [*ν*_2_ (^4^*A*_2*g*_→^4^*T*_1*g*_ (F))] and 615 nm [*ν*_1_ (^4^*A*_2*g*_→^4^*T*_2*g*_)] were the d-d transition absorption peaks of Cr(III) [35,36,37]. After forming the complex, the d-d transition absorption peaks of Cr(III) were 416 nm (*ε =* 7.11 × 10^3^ L·mol⁻^1^·cm⁻^1^) and 587 nm (*ε* = 4.82 × 10^3^ L·mol⁻^1^·cm⁻^1^), respectively. The absorption peaks were blueshifted at 13 nm and 28 nm, respectively. This was due to the effect of the hydroxyl group of the OBG on the 3d orbital of the trivalent chromium. An electron transition occurred, resulting in a blue shift. This indicated that the trivalent chromium formed a complex with the hydroxyl group of OBG.

#### 2.2.5. Circular Dichroism (CD) Spectrogram Analysis

**Figure 5:**
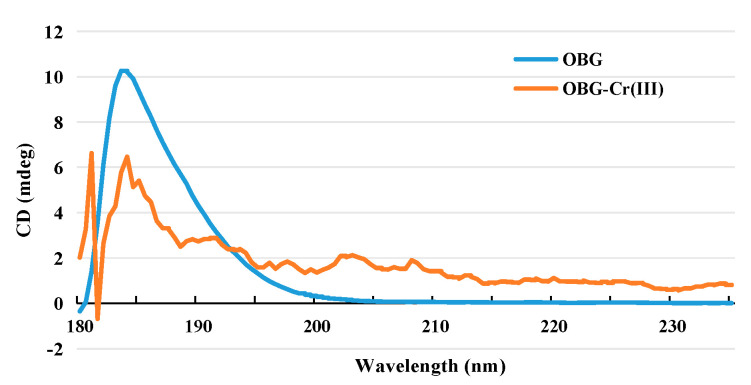
CD spectrogram of OBG and OBG-Cr(III).

CD spectroscopy is an effective method to study the spatial conformation of polysaccharides and can be used to determine the conformational changes of polysaccharides [38]. As can be seen from Figure 5, the circular dichroism (molecular asymmetry) of OBG in aqueous solution was weak. There was only one absorption peak at 184 nm, which was a positive Cotton effect. This was obviously related to the homogeneous molecular structure of oat *β*-glucan with a linear non-branched chain. After forming OBG-Cr(III), besides 184 nm, a positive Cotton effect appeared at 181 nm. The results showed that after the formation of the complex, the asymmetry of the molecule increased and the spatial conformation changed. This was related to the effect of Cr(III) on intramolecular and intermolecular hydrogen bonds after forming the complex with oat *β*-glucan.

#### 2.2.6. X-ray Powder Diffraction (XRD) Analysis

**Figure 6:**
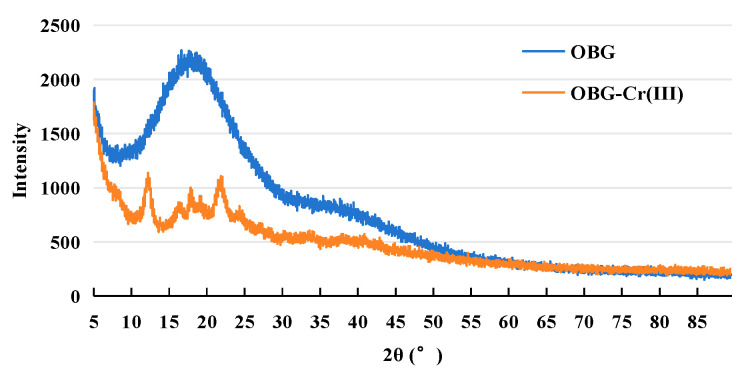
X-ray powder diffraction pattern of OBG and OBG-Cr(III).

The XRD patterns of OBG and OBG-Cr(III) are shown in Figure 6. The OBG had a strong broad peak at about 19°. This was similar to the diffraction results of other polysaccharides [24,26,27]. After the formation of the complex, the strength of this broad single peak was greatly attenuated and some of the acute diffraction peaks (12.3°, 16.6°, 18.0°, 19.3°, and 22.0°) appeared. The results showed that the crystal state of OBG clearly changed after the formation of OBG-Cr(III). We calculated the crystal particle size of OBG-Cr(III) from the powder X-ray according to the Scherrer equation [39,40,41]. The crystal particle size of OBG-Cr(III) was 9.06 nm. According to the powder X-ray, the OBG was in an amorphous powder state, so its crystal particle size was not tested. This resulted in the change in crystal morphology, which was consistent with the result of Section 2.2.7. The change in the crystal state was related to the coordination reaction with the hydroxyl groups of OBG after the formation of the Cr(III) complex, which could destroy the intramolecular and intermolecular hydrogen bonds and lead to the change in the crystal state.

#### 2.2.7. Scanning Electron Microscopy (SEM)–Energy Dispersive X-ray Spectroscopy (EDS) Analysis

**Figure 7:**
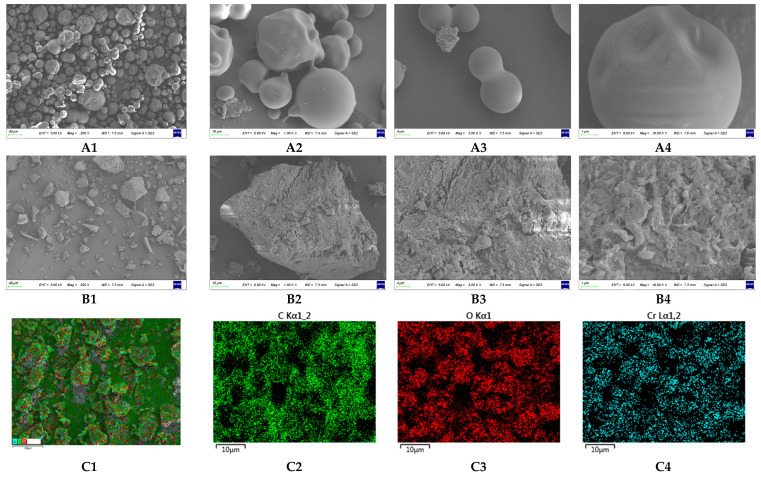

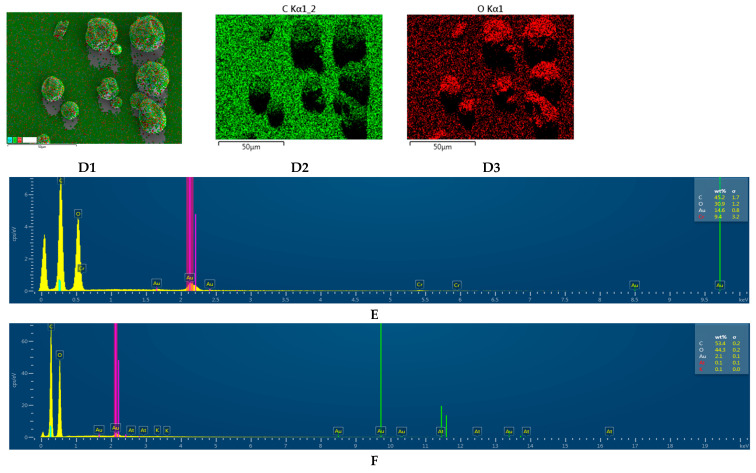
SEM-EDS analysis ((**A1**–**A4**) SEM images of OBG; (**B1**–**B4**) SEM images of OBG-Cr(III); (**C1**–**C4**) corresponding mapping images of OBG-Cr(III); (**D1**–**D3**) corresponding mapping images of OBG; (**E**) EDS spectra and the corresponding elemental contents of OBG-Cr(III); (**F**) EDS spectra and the corresponding elemental contents of OBG).

Figure 7(A1–A4) show the microscopic morphology of OBG. OBG was spherical and had a smooth surface. Figure 7(B1–B4) show the microscopic morphology of OBG-Cr(III). OBG-Cr(III) showed angular shape and the surface was not smooth. The results showed that Cr atoms entered into the interior of OBG molecules and reacted with hydroxyl groups, which resulted in the change in the crystal structure and different microscopic morphology. The results are consistent with those of XRD. The SEM-EDS elemental mapping images of C, O, and Cr were obtained by scanning OBG and OBG-Cr(III), as shown in Figure 7(C1–C4,D1–D3). As shown in Figure 7E,F, the EDS spectra for C and O had corresponding characteristic signal spectral peaks near 0.27 keV and 0.52 keV, respectively. Figure 7E shows that the OBG-Cr(III) had characteristic signal peaks for chromium at 0.57 keV, 5.41 keV, and 5.95 keV. The proportion of chromium in OBG-Cr(III) was 9.4%, which was basically consistent with the result of Section 2.2.2.

### 2.3. Determination of Inhibitory Activity against α-Amylase and α-Glucosidase

**Figure 8:**
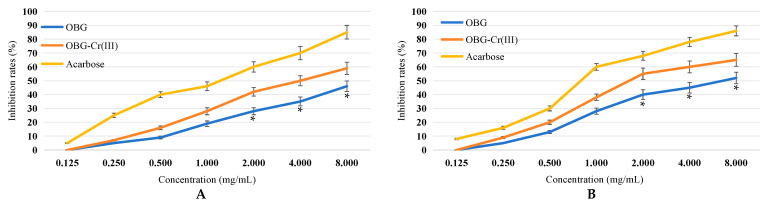
Inhibitory activity against α-amylase and α-glucosidase. (**A**) α-amylase inhibition rate. (**B**) α-glucosidase inhibition rate. The differences between the inhibition rates of OBG and OBG-Cr(III) were statistically significant (denoted by “*” for *p* < 0.05).

As shown in Figure 8A,B, both OBG and OBG-Cr(III) had inhibitory effects on both α-amylase and α-glucosidase. The inhibition rates of OBG and OBG-Cr(III) increased with the increase in concentration. The inhibitory activities of OBG-Cr(III) on α-amylase and α-glucosidase were better than that of OBG. At concentrations of 2, 4, and 8 mg/mL, the inhibitory activities of OBG-Cr(III) on α-amylase and α-glucosidase were significantly superior to that of OBG (*p* < 0.05). The IC_50_ of OBG and OBG-Cr(III) for α-amylase was calculated to be 9.87 ± 0.82 mg/mL and 4.68 ± 0.64 mg/mL, respectively. The results indicated that OBG-Cr(III) had a better inhibitory effect for α-amylase (*p* < 0.01). The IC_50_ of acarbose for α-amylase was calculated to be 0.98 ± 0.12 mg/mL. The results showed that the inhibitory effects of OBG-Cr(III) and OBG for α-amylase were less good than that of acarbose (*p* < 0.01). The IC_50_ of OBG and OBG-Cr(III) for α-glucosidase was 7.86 ± 0.73 mg/mL and 2.23 ± 0.12 mg/mL, respectively. The results indicated that OBG-Cr(III) had a better inhibitory effect for α-glucosidase (*p* < 0.01). The IC_50_ of acarbose for α-glucosidase was calculated to be 0.68 ± 0.07 mg/mL. The results showed that the inhibitory effects of OBG-Cr(III) and OBG for α-glucosidase were less good than that of acarbose (*p* < 0.01). These results suggested that the inhibitory activity of OBG on α-amylase and α-glucosidase increased greatly after the formation of the chromium complex, which was beneficial to the hypoglycemic activity.

### 2.4. Hypoglycemic Activity in IR-HepG2 Cells

#### 2.4.1. IR-HepG2 Cells Model Detection

**Figure 9:**
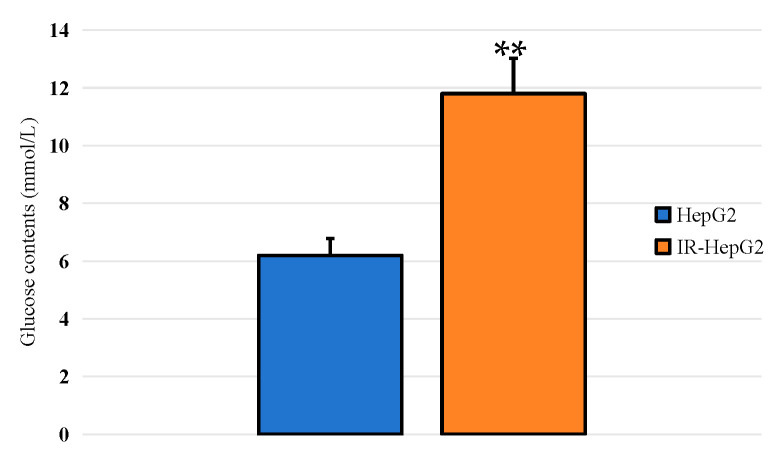
Detection of glucose consumption in IR-HepG2 cells. The difference between control and model group was statistically significant (denoted by “**” for *p* < 0.01).

As shown in Figure 9, after 36 h of culture, compared with normal HepG2 cells, the glucose content of IR model cells was significantly increased (*p* < 0.01). The results indicated that the glucose consumption of the cells was significantly reduced, and the IR-HepG2 cells model was formed.

#### 2.4.2. Effects on Cell Viability

**Figure 10:**
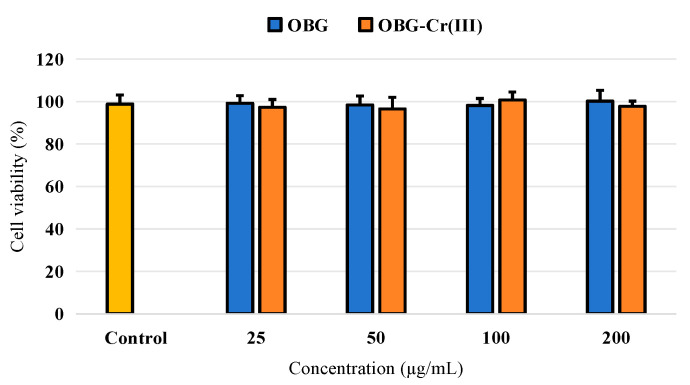
Effects on cell viability.

From Figure 10, compared with the control group, OBG and OBG-Cr(III) had no significant effect on HepG2 cells in the range of measured concentrations. These results suggested that OBG and OBG-Cr(III) had no obvious cytotoxicity to HepG2 cells.

#### 2.4.3. Effects on Glycometabolism in IR-HepG2 Cells

**Figure 11:**
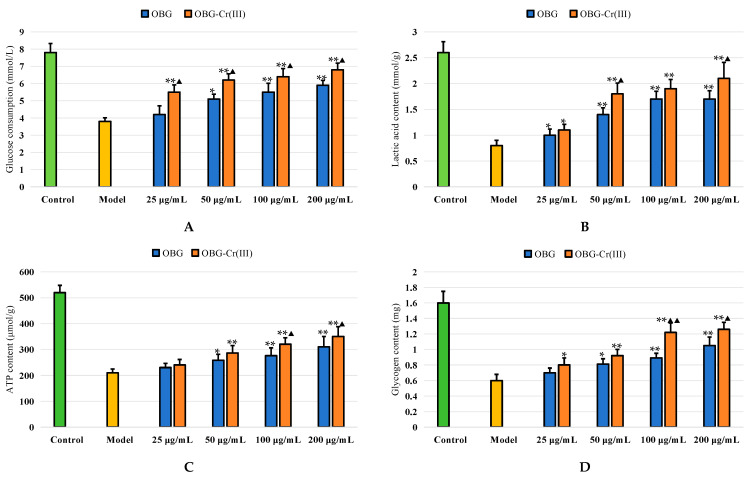
Hypoglycemic activities of OBG and OBG-Cr(III) in IR-HepG2 cells. Glucose consumption (**A**); lactic acid content (**B**); ATP content (**C**); glycogen content (**D**). The differences between these groups and the model group were statistically significant (denoted by “*” for *p* < 0.05, “**” for *p* < 0.01); the differences between the OBG group and the OBG-Cr(III) group were statistically significant (denoted by “^▲^” for *p* < 0.05, “^▲▲^” for *p* < 0.01).

As shown in Figure 11, IR-HepG2 cells in the model group had significantly lower glucose consumption than normal HepG2 cells in the control group. This indicated that the model of IR-HepG2 cells was successful. The glucose consumption of OBG and OBG-Cr(III) group increased with the increase in concentration. The glucose consumption of OBG and OBG-Cr(III) in different dose groups was significantly increased compared with the model group (*p* < 0.05 or *p* < 0.01). Compared with the model group, the contents of lactic acid, ATP, and glycogen in OBG and OBG-Cr(III) groups were significantly improved (*p* < 0.05 or *p* < 0.01). Efficacy was positively correlated with the dose. More importantly, OBG-Cr(III) had superior activity to OBG in regulating cellular metabolism (*p* < 0.05 or *p* < 0.01). This indicated that OBG and OBG-Cr(III) had good hypoglycemic activity. Compared with OBG, different concentrations of OBG-Cr(III) had better hypoglycemic activity. It was concluded that the chromium complex had a positive effect on the glucose-lowering activity of OBG.

#### 2.4.4. OBG and OBG-Cr(III) Regulated the Expression of c-Cbl/PI3K/AKT/GLUT4 in IR-HepG2 Cells

**Figure 12:**
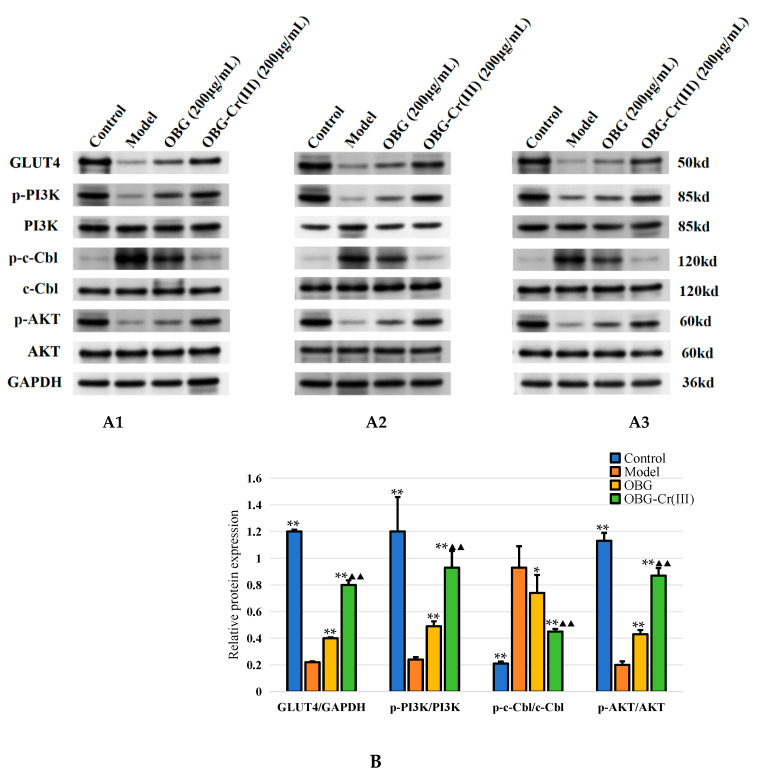
Effects of OBG and OBG-Cr(III) on the expression of related proteins in IR-HepG2 cells. Western blot bands of p-c-Cbl, p-PI3K, p-AKT, and GLUT4 ((**A1**–**A3**), three biological replicates); protein expression levels of GLUT4/GAPDH, p-PI3K/PI3K, p-c-Cbl/c-Cbl, and p-AKT/AKT (**B**). The differences between these groups and the model group were statistically significant (denoted by “*” for *p* < 0.05, “**” for *p* < 0.01); the differences between the OBG group and the OBG-Cr(III) group were statistically significant (denoted by “^▲▲^” for *p* < 0.01).

The PI3K/AKT pathway is a classical insulin signaling pathway, and the activation of this pathway can promote insulin signaling and increase insulin sensitivity [42]. The phosphorylation of AKT can affect the expression and membrane translocation of its downstream protein GLUT4, and this process can promote the uptake of glucose into the cell transport process, thus playing a hypoglycemic role [43]. Therefore, the detection of insulin signaling pathway and glucose transport-related protein changes can reflect the role of drugs to improve IR. As shown in Figure 12, compared with the control group, the relative expression levels of GLUT4/GAPDH, p-PI3K/PI3K, and p-AKT/AKT in the model group were significantly decreased (*p* < 0.01), and the relative expression level of p-c-Cbl/c-Cbl was significantly increased (*p* < 0.01). Compared with the model group, the relative expression levels of GLUT4/GAPDH, p-PI3K/PI3K, and p-AKT/AKT in the OBG group and OBG-Cr(III) group were significantly increased (*p* < 0.01), and the relative expression levels of p-c-Cbl/c-Cbl were significantly decreased (*p* < 0.05 or *p* < 0.01). The results showed that the OBG group and OBG-Cr(III) group could improve insulin resistance by regulating c-Cbl/PI3K/AKT/GLUT4 expression. The effect of the OBG-Cr(III) group was better than that of the OBG group (*p* < 0.01). This was consistent with the better hypoglycemic activity of OBG-Cr(III) in IR-HepG2 cells, which indicated that Cr(III) and OBG could form a chromium complex with synergistic effect.

## 3. Discussion

In this study, we selected OBG as the ligand due to its clear hypoglycemic activity. In 1977, the US FDA stated that consuming oatmeal foods over a certain period and reaching the oat *β*-glucan threshold could lead to hypoglycemic effects [17]. Subsequent research confirmed the hypoglycemic activity of OBG [44,45,46,47]. Another advantage of OBG is its ability to be derived from bulk grains, making it more readily available. Considering these factors, OBG-Cr(III) showed potential as an auxiliary hypoglycemic functional factor for reducing diabetes incidence.

During the synthesis of OBG-Cr(III), various conditions, such as temperature, pH, and material ratio, were explored. Suitable reaction conditions were identified, resulting in the successful synthesis and purification of OBG-Cr(III). It was observed that different pH levels significantly influenced the yield and chromium content of OBG-Cr(III). In combination with our other studies, we also found that different polysaccharides had different optimal pH values to promote the reaction and affect the chromium contents of the complexes. The structural analysis of OBG-Cr(III) using various methods indicated significant changes in spatial conformation, external morphology, and crystalline state compared to OBG, which were consistent with findings from other studies [48,49,50]. These changes were attributed to hydrogen bonding between Cr and OBG, potentially contributing to improved hypoglycemic activity post complex formation.

Both OBG and OBG-Cr(III) exhibited potent inhibitory activities against *α*-amylase and *α*-glucosidase, with OBG-Cr(III) demonstrating superior activity. The enhanced activity may be attributed to alterations in spatial conformation post complex formation and the influence of chromium. Further research is needed to confirm these findings.

IR is often associated with aberrant glucose metabolism. To assess the hypoglycemic potential of OBG-Cr(III), we utilized an insulin-resistant HepG2 (IR-HepG2) cell model. Neither OBG nor OBG-Cr(III) affected cell activity, confirming their safety profile. Compared with OBG treatment alone, the OBG-Cr(III) intervention led to increased levels of glucose consumption, lactic acid, ATP, and glycogen in IR-HepG2 cells, indicating enhanced glucose metabolism. This showed that chromium and OBG form a complex which could play a synergistic role in hypoglycemic activity. To elucidate the underlying mechanism, we investigated IR-related signaling pathways. c-Cbl is an important adaptor protein involved in the energy balance process in the body. c-Cbl is thought to be an E3 ubiquitin ligase with a characteristic RING Finger (RNF) domain that regulates a variety of receptor-mediated signals on cell membrane surfaces [51,52]. c-Cbl plays a vital role in regulating insulin receptor tyrosine kinase signaling and is considered a potential therapeutic target for type 2 diabetes and obesity [53]. The knockout of the c-Cbl gene has been shown to ameliorate high-fat diet-induced obesity and insulin resistance in diabetic mouse models [54]. The PI3K/AKT signaling pathway, which is closely linked to IR-related disorders, is activated upon insulin stimulation, leading to the upregulation of relevant molecules and subsequent GLUT4 activation and translocation to the cell membrane for glucose uptake [55,56]. Reduced GLUT4 expression or activity is a key molecular feature of IR [57]. Thus, modulating the expression of c-Cbl/PI3K/AKT signaling pathway-related molecules to facilitate GLUT4 translocation to the cell membrane has emerged as a therapeutic approach for IR-related conditions. Our study revealed alterations in the protein expression of p-c-Cbl, p-PI3K, p-AKT, and GLUT4 in IR-HepG2 cells, which were reversed by the OBG-Cr(III) complex. Importantly, the effects of OBG-Cr(III) were superior to those of OBG alone. Consequently, the complex enhanced insulin sensitivity in IR-HepG2 cells and promoted intracellular glucose metabolism by regulating the expression of the c-Cbl/PI3K/AKT signaling pathway.

According to the inhibitory activity of *α*-glucosidase and *α*-amylase, and the results of hypoglycemic activity in IR-HepG2 cell model, OBG and OBG-Cr(III) showed different hypoglycemic activity. OBG-Cr(III) showed better hypoglycemic activity than OBG in enzyme inhibition activity and the IR-HepG2 cell model. The improvement in hypoglycemic activity might be related to their structural characteristics, including molecular weight, monosaccharide composition, glycosidic linkage, conformation, branching degree, and functional groups [58,59]. We analyzed their structure–activity relationship.

OBG is a linear polysaccharide of D-glucose linked by *β* (1→4) and *β* (1→3) glycosidic bonds [38]. By introducing Cr(III) into OBG, the structure of OBG clearly changed. The molecular asymmetry and spatial structure of OBG-Cr(III) were quite different from those of linear OBG. These changes in structure may result in a more extensive and stronger binding of OBG-Cr(III) to *α*-glucosidase and *α*-amylase binding sites. The introduction of Cr(III) may further increase the binding of OBG-Cr(III) to the enzyme. These effects led to better enzyme inhibitory activity of the complex. Cui et al. [60] showed that lentinula edodes *β*-glucan and yeast *β*-glucan had a more complex trihelix structure, which could form stronger hydrogen bonds with starch granules compared with OBG without trihelix structure. This further proved that the linear OBG transformed into OBG-Cr(III) with a complex spatial structure, which was beneficial to the activity enhancement. Xu et al. [61] reported that the polysaccharide from blackcurrant fruits with a lower molecular weight exhibited higher α-glucosidase inhibitory. The molecular weight of OBG was 8.894 × 10^4^ Da, and the molecular weight of OBG-Cr(III) was 7.736 × 10^4^ Da. The decrease in molecular weight was also beneficial to the improvement of enzyme inhibitory activity.

The conversion of OBG to OBG-Cr(III) showed no significant changes in monosaccharide composition, glycosidic linkage, or functional groups. When OBG was transformed into OBG-Cr(III), the main change in structure concerned spatial structure. The linear OBG structure was transformed into the twisted and complicated OBG-Cr(III) structure. This structural alteration enabled OBG-Cr(III) to better regulate the c-Cbl/PI3K/AKT/GLUT4 signaling pathway. In addition, the introduction of Cr(III) was also essential to improve IR. In a previous study, sulfated rhamnose polysaccharides enhanced glucose metabolism after the introduction of Cr(III) by modulating insulin-mediated PI3K/PKB/GSK-3β [24]. These results indicated that Cr(III) in OBG-Cr(III) had a strong hypoglycemic activity. Molecular weight was also an important factor affecting the hypoglycemic activity of polysaccharides. Molecular weight can affect solubility, digestibility, metabolic rate and bioavailability, and low-molecular-weight polysaccharides can be absorbed by cells and the intestinal tract into the blood [62]. In this study, OBG-Cr(III) had a better hypoglycemic activity, which may be due to the low molecular weight and spatial structure changes which made it easier for OBG-Cr(III) to enter cells. Low molecular weight is also beneficial to the increase in solubility, and high solubility is more likely to repair the surface damage of IR-HepG2 cells.

## 4. Materials and Methods

### 4.1. Materials and Reagents

Oat *β*-glucan was obtained from Hebei Minsheng Food Ingredients Co., Ltd. (Langfang, China, purity ≥ 90%). CrCl_3_·6H_2_O was obtained from Shanghai ZZBIO Co., Ltd. (Shanghai, China, purity ≥ 99%). NaOH (purity ≥ 99%) was obtained from Tianjin Beichen Fangzheng reagents Co., Ltd. (Tianjin, China). α-amylase (50 U/mg), α-glucosidase (50 U/mg), and acarbose (purity ≥ 98%) were obtained from Shanghai Macklin Biochemical Technology Co., Ltd. (Shanghai, China). HepG2 cells were acquired from The Chinese Academy of Sciences Shanghai Cell Bank, Shanghai, China. Metformin (purity ≥ 97%) was obtained from Shanghai Aladdin Bio-chemical Technology Co., Ltd. (Shanghai, China). Insulin was obtained from Sigma-Aldrich Co., Ltd. (St. Louis, MO, USA). DMEM was obtained from Wuhan Servicebio Technology Co., Ltd. (Wuhan, China). Fetal bovine serum was obtained from Zhejiang Tianhang Biotechnology Co., Ltd. (Huzhou, China). Dimethyl sulfoxide was obtained from Sigma-Aldrich Co., Ltd. (St. Louis, MO, USA). Glucose, lactic acid, adenosine triphosphate (ATP), and glycogen assay kits were obtained from Nanjing Jiancheng Bioengineering Institute (Nanjing, China). The c-casitas B-lineage lymphoma (c-Cbl) antibody and p-c-Cbl antibody (Nanjing Jiancheng Bioengineering Institute, Nanjing, China), phosphatidylinositol 3-kinase (PI3K) antibody and p-PI3K antibody (BIOSS, Beijing, China), protein kinase B (AKT) antibody and p-AKT antibody (Cell Signaling Technology, Danvers, MA, USA), glucose transporter 4 (GLUT4) antibody (Affinity Biosciences, Cincinnati, OH, USA), and glyceraldehyde-3-phosphate dehydrogenase (GAPDH) antibody (Nanjing Jiancheng Bioengineering Institute, Nanjing, China) were used. Anti-rabbit immunoglobulin (Ig)G antibody was purchased from Amertek Technology (Berwyn, MA, USA). Chemiluminescence reagents and polyvinylidene difluoride (PVDF) membranes were purchased from Millipore (Billerica, MA, USA). All chemicals and solvents used were of analytical or high-performance liquid chromatography grade.

### 4.2. Preparation of the Oat β-Glucan–Chromium(III) Complex [OBG-Cr(III)]

A total of 3 g oat *β*-glucan was dissolved in 50 mL deionized water. A 2 mol·L^−1^ CrCl_3_·6H_2_O solution and a 2 mol·L^−1^ NaOH solution were prepared with deionized water.

Under the condition of 70 °C, 50 mL oat *β*-glucan solution was adjusted to pH = 8. In the absence of precipitation, the CrCl_3_·6H_2_O solution and the NaOH solution were slowly added to maintain pH = 7~8. A drop of the CrCl_3_·6H_2_O solution was added until flocculation appeared in the solution and then the reaction was maintained at 70 °C for 2 h. After the reaction, the solution was cooled to room temperature, centrifuged at 3000× *g* for 5 min, and the supernatant was obtained. Three times the volume of anhydrous ethanol was added into the supernatant, and the precipitate was collected by means of centrifugation at 3000× *g*. The crude oat *β*-glucan–chromium (III) complex was obtained by washing the precipitate with anhydrous ethanol, acetone, and ether, and drying under reduced pressure.

Dialysis bag processing: The dialysis bag was boiled in a mixture of 2% NaHCO_3_ and 1 mmol/L EDTA (pH = 8) for 10 min, and rinsed with distilled water. After that, 1 mmol/L EDTA (pH = 8) was used to boil for 10 min, and cooled at 4 °C standby. The dialysis bag was guaranteed to be immersed in the preservation solution after treatment.

The above oat *β*-glucan–chromium(III) complex was dissolved in 50 mL deionized water. The solution was put into the dialysis bag for dialysis overnight. The crude oat *β*-glucan–chromium(III) complex was obtained through concentration after dialysis. Finally, it was purified using Sephadex gel column chromatography (deionized water was used as an eluent).

### 4.3. Characterization of OBG-Cr(III)

#### 4.3.1. Molecular Weight Determination of OBG and OBG-Cr(III)

The molecular weights of OBG and OBG-CR (III) were determined using high-performance gel permeation chromatography. The instrument used was Agilent PL-GPC-50 system (Agilent Technologies, Santa Clara, CA, USA). The concentrations of all samples were 2 mg/mL. The chromatographic separation conditions were as follows: the column was a PL aquagel-OH column (7.5 × 50 mm, 8 μm and 7.5 × 300 mm, 8 μm, Agilent Technologies, Santa Clara, CA, USA), the detector was a refractive index detector, the mobile phase was 0.2 mol/L NaNO_3_ solution, the column temperature was 40 °C, the flow rate was 1 mL/min, and the injection volume was 100 μL.

#### 4.3.2. Chromium Content Determination of OBG-Cr(III)

The content of chromium in OBG-Cr(III) was determined using an inductively coupled plasma mass spectrometer (ICP-MS, Agilent 7700, Agilent Technologies, USA). A total of 0.05 g OBG-Cr(III) was added into the digestive tube and digested with 2 mL of 50% nitric acid for 15 min. The tube was then placed in a graphite digester and heated at a preset programmed temperature (room temperature ~80 °C 10 min, 80 °C~150 °C 10 min, 150 °C~200 °C 3 min, and 200 °C 30 min). After digestion, the solution was cooled to room temperature, filtered, and fixed to 10 mL with deionized water. The content of chromium in the complex was determined by means of ICP-MS.

#### 4.3.3. FT-IR Spectroscopy Determination

A suitable amount of OBG and OBG-Cr(III) sample powders were placed on the sample stage of the Fourier-transform infrared spectrometer (Nicolet iS5 FT-IR, Thermo Fisher Scientific Inc., Waltham, USA) for scanning, and the background interference was deducted. The scanning range was from 4000 cm^−1^ to 400 cm^−1^, and the scanning resolution was set at 4 cm^−1^. The characteristic absorption of each functional group was analyzed.

#### 4.3.4. UV-VIS Spectroscopy Determination

OBG-Cr(III) was prepared into 8 mg/mL deionized water solution, and the deionized water was used as reference. The UV-Vis spectra were measured using a UV-Vis spectrometer (TU-1810PC UV-Visible Spectrophotometer, Persee Instruments Co., Beijing, China). The scanning range was 190–800 nm, and the scanning interval was 0.5 nm. A CrCl_3_•6H_2_O deionized aqueous solution (10mg/mL) was prepared, and the UV-Vis spectra of CrCl_3_•6H_2_O were determined and compared with those of the chromium complex.

#### 4.3.5. CD Determination

The conformational changes of OBG and OBG-Cr(III) were analyzed using a CD spectrometer (J-1500 CD Spectrophotometer, JASCO, Tokyo, Japan). The concentrations of all the samples were 1 mg/mL, and the measurement range was set at 180~400 nm. The scanning speed was set at 100 nm/min, with a bandwidth of 2.0 nm, and the time constant was 2 s.

#### 4.3.6. XRD Determination

The crystalline states of OBG and OBG-Cr(III) were determined using an X-ray powder diffractometer (D8 ADVANCE, Bruker Corporation, Karlsruhe, Germany). The conditions for the XRD analysis were as follows: the voltage was set at 40 kV, the current was set at 40 mA, the scanning range θ = 5~90° (θ was the angle of diffraction), and the scanning speed was set at 5°/min.

#### 4.3.7. SEM-EDS Determination

The microscopic morphology of OBG and OBG-Cr(III) was studied using SEM (Zeiss Sigma 500, Carl Zeiss AG, Oberkochen, Germany), and the element contents were analyzed using EDS. The dried sample powder was evenly spread on the double-sided conductive adhesive and fixed on the aluminum sample plate. Gold was sprayed in a vacuum spray plating apparatus, and sample images were observed and recorded under high vacuum at an accelerating voltage of 5.0 kV. The microscopic morphology of the samples was then examined at magnifications of 200-, 1000-, 2000-, and 10,000-fold.

### 4.4. Inhibitory Activity against α-Amylase and α-Glucosidase [27]

#### 4.4.1. Inhibitory Activity against α-Amylase

The samples were prepared into solutions with the concentrations of 0.125, 0.250, 0.500, 1.000, 2.000, 4.000, and 8.000 mg/mL, and 1 mL of α-amylase solution (2 U/mL) was added into 1 mL sample solutions, preheated in a 37 °C water bath. At the same time, the starch solution (1%) was preheated at the same temperature, and after 5 min, 0.3 mL of starch solution was added. After 5 min, 0.5 mL of 3, 5-dinitrosalicylic acid solution was added and the mixture was heated in boiling water bath for 5 min. After cooling to room temperature, distilled water was added to the solution for 5 mL, and the absorbance value was determined at the wavelength of 540 nm (*A*_3_). The absorbance value (*A*_1_) was determined using distilled water instead of samples, and the other operations were the same. Using distilled water instead of alpha-amylase and samples, the other operations were the same, and the absorbance value (*A*_2_) was determined. Using distilled water instead of alpha-amylase, the other operations were the same and the absorbance value (*A*_4_) was determined. In the positive control group, acarbose was used instead of sample solutions. The formula of the inhibition rate is shown as Formula (1).

(1) $$\alpha -amylase\;inhibition\;\mathrm{rate}\;(\%)=\left(1-\frac{A_{3}-A_{4}}{A_{1}-A_{2}}\right)\times 100$$

#### 4.4.2. Inhibitory Activity against α-Glucosidase

The samples were prepared into solutions with the concentrations of 0.125, 0.250, 0.500, 1.000, 2.000, 4.000, and 8.000 mg/mL. A total of1 mL solution was added with 1 mL α-glucosidase (2 U/mL). Then, 2 mL of PBS buffer solution (pH = 6.8) was added and the reaction time was 10 min at 37 °C; then, 0.1 mL of glutathione solution (3 mmol/L) and 0.25 mL of p-nitrophenyl-*β*-D-galactosyl-pyranoside (10 mmol/L) were added at the same temperature, and the reaction time was 10 min. Finally, 2 mL sodium carbonate solution (0.1 mol/L) was added and the reaction was terminated. The absorbance value (*A*) was measured at 400 nm. The absorbance value (*A*_0_) was determined by using PBS buffer solution instead of sample solutions. The absorbance value (*A*_1_) was determined by using PBS buffer solution instead of α-glucosidase solution. The absorbance value (*A*_2_) was determined by using PBS buffer solution instead of α-glucosidase solution and sample solutions. In the positive control group, acarbose was used instead of sample solutions. The formula of inhibition rate is shown as Formula (2).

(2) $$\alpha -glucosiade\;inhibition\;rate\;(\%)=\frac{\left(A_{0}-A_{2})-(A-A_{1}\right)}{\left(A_{0}-A_{2}\right)}\times 100$$

### 4.5. Study on Improving Insulin Resistance of HepG2 Cells

#### 4.5.1. HepG2 Cell Culture

HepG2 cells were thawed from liquid nitrogen and subcultured. The HepG2 cells were resuscitated in a 37 °C water bath. The cells were centrifugally precipitated and then put into a cell culture dish containing 89% high-glucose DMEM medium, 10% fetal bovine serum, and 1% penicillin–streptomycin mixture on a super-clean table. The cells were cultured in incubators (at 37 °C with 5% CO_2_). When the cells were at 80% confluence growth, the cells were washed with PBS, the adherent cells were digested with 0.25% trypsin, and then the digestion of cells was stopped with serum-containing medium. The cells were transferred into a centrifuge tube and centrifuged at 1500 r/min. After centrifugation, the upper medium was removed and new medium was added and was then mixed so that it became a uniform cell suspension. The cell suspension was divided into new culture flasks for further culture.

#### 4.5.2. HepG2 Cell Viability Assay

The HepG2 cells in logarithmic growth phase were obtained. After dilution, 1 × 10^5^ cells/mL were counted under the microscope. The cells were seeded into 96-well culture plates with 100 μL cell suspension per well. After culture for 24 h, 100 μL different concentrations (25, 50, 100, and 200 μg/mL) of sample solutions were added. After culture for 24 h, 50 μL MTT solution (1 mg/mL) was added into each well and incubated in the dark for 4 h. The solution was removed from the culture well, and 100 μL DMSO was added to dissolve formazan. The absorbance of the solution was determined at 570 nm. The cell viability was calculated according to Formula (3).

(3) $$Cell\;viability(\%)=\frac{Asample-Ablank1}{Acontrol-Ablank2}\times 100$$

where *A_sample_* is the absorbance of the group with samples, *A_blank1_* is the absorbance of the sample group without cells, *A_control_* is the absorbance of the group without samples, and *A_blank2_* is absorbance of the control group without cells.

#### 4.5.3. Hypoglycemic Activity in IR-HepG2 Cells

The HepG2 cells at logarithmic growth stage were washed with PBS solution and digested with 0.25% trypsin, and then a medium was added to stop digestion. After centrifugation, the original medium was discarded and a new medium was added and mixed into the cell suspension. The cells were seeded in 96-well culture plates with 6 repeated wells in a row, and the original medium was discarded after 24 h of culture. An insulin-resistant cell model was established by adding 100 μL medium to 10^−6^ mol/L insulin and culturing for 36 h. The cell model was starved with serum-free medium and washed with PBS after 12 h of culture. Then, 200 μL of OBG and OBG-Cr(III) medium solutions (25, 50, 100, and 200 μg/mL) were added to each well and then cultured for 24 h. The control group were HepG2 cells and the model group were IR-HepG2 cells. According to the instructions of the glucose assay kit, the OD values of each group were measured at 450 nm wavelength in an enzyme-labeled instrument, and the glucose consumption was calculated. The contents of ATP, lactic acid, and glycogen were measured using ATP, lactate, and glycogen assay kits.

#### 4.5.4. Western Blot Analysis

The method of Western blot was moderately modified from the reported method [63,64]. The expression of GLUT4, c-Cbl, PI3K, and Akt protein were detected after 24 h treatment. Total protein was extracted and quantified using the method of BCA. The protein was separated by means of SDS-PAGE electrophoresis. The proteins were transferred to a PVDF membrane using the wet transfer method and were blocked with 5% skim milk powder in TBST buffer at room temperature for 2 h. Diluted primary antibodies (GLUT4: 1:1000; c-Cbl: 1:1000; p-c-Cbl: 1:1000; PI3K: 1:2000; p-PI3K: 1:1000; AKT: 1:500; p-AKT: 1:1000; and GAPDH: 1:2000) were added, and the membrane was rinsed using TBST after incubation at room temperature for 1 h. The secondary antibodies dilutions (1:10,000) were added, and the mixture was incubated for 1h at room temperature, and rinsed with TBST. After the addition of a chemiluminescence reagent, exposure, and development in the dark room, the gray value of each protein band was read.

### 4.6. Statistical Analysis

The data are given as means ± SEMs. Comparisons between experimental groups were performed using one-way ANOVA, followed by Student–Newman–Keuls tests. *p* values < 0.05 or <0.01 were considered to indicate significant differences or extremely distinct differences, respectively (SPSS (PASW) 20.0).

## 5. Conclusions

In this study, a novel oat *β*-glucan–chromium(III) complex (OBG-Cr(III)) was synthesized by chelating oat *β*-glucan with chromium(III). The structural characterization revealed that the content of chromium in OBG-Cr(III) was 10.87%, and chromium(III) was bound to the hydroxyl group of oat *β*-glucan. The introduction of chromium(III) also led to changes in the spatial conformation, external morphology, and crystal state of oat *β*-glucan. Compared with OBG, OBG-Cr(III) exhibited a better inhibitory activity against α-amylase and α-glucosidase and a better hypoglycemic activity for IR-HepG2 cells. The Western blot analysis showed that OBG-Cr(III) could improve the expression of p-c-Cbl, p-PI3K, p-AKT, and GLUT4. These results suggested that OBG-Cr(III) played a role in hypoglycemic activity by regulating the c-Cbl/PI3K/AKT/GLUT4 signaling pathway. The hypoglycemic activity of OBG-Cr(III) was stronger than that of OBG due to the introduction of chromium(III) and the change in the spatial conformation of OBG. Certainly, the in-depth mechanism of the hypoglycemic activity of OBG-Cr(III) needs to be further investigated through animal models to study its effects on the gut microbiota and intestinal absorption. In conclusion, a Cr(III) complex can improve the glucose-lowering biological activity of OBG, which is valuable for expanding the application of OBG and developing glucose-lowering supplements in functional foods.

## Figures and Tables

**Table 1:** Chromium content analysis of OBG-Cr(III).

Test Items	Values
Sample Quality m_0_ (g)	0.0412
Volume V_0_ (mL)	10
Test Elements	Cr
Element Concentration of Test Solution C_0_ (mg/L)	8.9541
Dilution Ratio f	50
Element Concentration of Digestion Solution C_1_ (mg/L)	447.7025
Sample Element Content C_x_ (mg/kg)	108,665.66
Sample Element Content W	10.8666%

## Data Availability

Data are available in the manuscript.
